# Enrichment allows identification of diverse, rare elements in metagenomic resistome-virulome sequencing

**DOI:** 10.1186/s40168-017-0361-8

**Published:** 2017-10-17

**Authors:** Noelle R. Noyes, Maggie E. Weinroth, Jennifer K. Parker, Chris J. Dean, Steven M. Lakin, Robert A. Raymond, Pablo Rovira, Enrique Doster, Zaid Abdo, Jennifer N. Martin, Kenneth L. Jones, Jaime Ruiz, Christina A. Boucher, Keith E. Belk, Paul S. Morley

**Affiliations:** 1 Department of Microbiology, Immunology and Pathology, Colorado State University, Fort Collins, CO USA; 2 Department of Animal Sciences, Colorado State University, Fort Collins, CO USA; 3 Department of Clinical Sciences, Colorado State University, Fort Collins, CO USA; 4 Department of Computer Sciences, Colorado State University, Fort Collins, CO USA; 5 Department of Pediatrics, Section of Hematology Oncology and Bone Marrow Transplant, University of Colorado School of Medicine, Aurora, CO USA; 6 Department of Computer and Information Science and Engineering, University of Florida, Gainesville, Florida, USA

**Keywords:** Resistome, Antimicrobial resistance, Molecular enrichment, Microbial ecology, Rare microbiome

## Abstract

**Background:**

Shotgun metagenomic sequencing is increasingly utilized as a tool to evaluate ecological-level dynamics of antimicrobial resistance and virulence, in conjunction with microbiome analysis. Interest in use of this method for environmental surveillance of antimicrobial resistance and pathogenic microorganisms is also increasing. In published metagenomic datasets, the total of all resistance- and virulence-related sequences accounts for < 1% of all sequenced DNA, leading to limitations in detection of low-abundance resistome-virulome elements. This study describes the extent and composition of the low-abundance portion of the resistome-virulome, using a bait-capture and enrichment system that incorporates unique molecular indices to count DNA molecules and correct for enrichment bias.

**Results:**

The use of the bait-capture and enrichment system significantly increased on-target sequencing of the resistome-virulome, enabling detection of an additional 1441 gene accessions and revealing a low-abundance portion of the resistome-virulome that was more diverse and compositionally different than that detected by more traditional metagenomic assays. The low-abundance portion of the resistome-virulome also contained resistance genes with public health importance, such as extended-spectrum betalactamases, that were not detected using traditional shotgun metagenomic sequencing. In addition, the use of the bait-capture and enrichment system enabled identification of rare resistance gene haplotypes that were used to discriminate between sample origins.

**Conclusions:**

These results demonstrate that the rare resistome-virulome contains valuable and unique information that can be utilized for both surveillance and population genetic investigations of resistance. Access to the rare resistome-virulome using the bait-capture and enrichment system validated in this study can greatly advance our understanding of microbiome-resistome dynamics.

**Electronic supplementary material:**

The online version of this article (10.1186/s40168-017-0361-8) contains supplementary material, which is available to authorized users.

## Background

The use of shotgun metagenomic sequencing to study antimicrobial resistance (AMR) has increased dramatically over the past several years. This approach enables characterization of all AMR genes within a microbial community (the “resistome”), which can be useful in understanding evolutionary shifts in AMR [1], as well as for detecting transfer of diverse AMR genes between hosts, environments, or uncultivable organisms [2]. AMR is an inherently ecological phenomenon, with processes including transfer of genetic elements between divergent bacteria, increased promiscuity and mutation in the face of bacterial stress and inflammation, and co-selection and co-mobility of multiple genes [3–6]. Shotgun metagenomics represents a tool for advancing our understanding of these interactions by enabling access to the genetic material of the microbial population as a whole [7]. In addition, metagenomic sampling could augment epidemiological surveillance and outbreak investigations of AMR [8, 9].

However, a central challenge of this approach lies in the fact that the resistome comprises a small proportion of all DNA in a metagenomic sample [10]. In previously published fecal metagenomic datasets sampled to a depth of ~ 100 M reads, fewer than 100,000 reads are typically attributed to the resistome [10, 11], meaning that > 99% of sequences could be considered “off-target” if the resistome is the primary study interest. This is of particular concern for epidemiological AMR surveillance efforts, which aim to detect AMR genes in large numbers of continuously collected samples and therefore cannot tolerate cost inefficiencies. Furthermore, effective AMR surveillance schemes must focus on AMR genes and AMR transfer events relevant to public health. Recent evidence suggests that such AMR genes (e.g., extended-spectrum betalactamases) are not necessarily the high-abundance resistome members and that horizontal gene transfer from environmental to clinical environments is a rare event [2, 12]. Therefore, even deep sequencing of metagenomic samples may not allow reliable capture of elements or events in the rarest portion of the resistome [12]. Aside from epidemiological surveillance, the existence and importance of rare or low-abundance members in the microbiome have been demonstrated clinically and ecologically [13, 14], prompting questions about whether the same dynamics exist within the resistome. However, very little has been described regarding the low-abundance portion of the resistome, perhaps because it is difficult to access.

Methods do exist for selectively depleting unwanted DNA from metagenomic samples prior to sequencing [15]. However, these methods are designed for depletion of eukaryotic content based on DNA/RNA characteristics that differ between eukaryotes and prokaryotes. Fecal, soil, and water samples do not typically contain large amounts of eukaryotic DNA but rather are dominated by bacterial DNA [11]. Recent advances show promise in depleting unwanted DNA based on sequence alone, and these methods could theoretically be applied to microbial sequences [16]. However, in metagenomic samples the unwanted DNA is comprised of thousands of bacterial species, many with unknown genomes. Alternatively, methods exist for proportional enrichment of wanted versus unwanted DNA. One such method is so-called bait-capture or target enrichment, an approach based on hybridization of pre-designed 120-mer biotinylated cRNA baits to target DNA for capture and subsequent enrichment [17]. Originally used for capture and sequencing of the human exome, this approach has been expanded to eukaryotic and pathogenic bacterial genomes [18, 19]. The ability to capture genetic variation is a major advantage of this approach over PCR, as was demonstrated recently for capturing the virome within metagenomic samples [20, 21]. Given these successes, one aim of this project was to determine whether the bait-capture and enrichment approach could be applied to resistance genes within a metagenomic sample.

However, bait-capture and enrichment have the potential to introduce bias into the metagenomic workflow, both through differential capture affinity and amplification rates. Many resistome-related analyses require quantification of resistome-related sequences, i.e., either absolute or relative abundances. Such quantification would be precluded by significant amounts of capture and/or amplification bias. Therefore, another aim of this work was to incorporate the use of unique molecular indices (UMIs) into the workflow [22]. These randomly generated 12-mer oligonucleotide sequences are affixed to individual DNA molecules prior to bait-capture and enrichment and thus can be used to correct for PCR bias and to count rare individual DNA sequences in post-sequencing analysis [23, 24].

To test the accuracy and efficiency of a combined bait-capture enrichment and UMI system (which we term MEGaRICH), 4 aliquots of whole-sample DNA from each of 16 samples were subjected to the following library preparation assays: (1) non-enriched metagenomic DNA libraries (“Metagenome”), (2) resistome-enriched metagenomic DNA libraries (“Resistome”), (3) non-enriched metagenomic DNA libraries with UMIs (“Metagenome-UMI”), and (4) resistome-enriched metagenomic DNA libraries with UMIs (“Resistome-UMI”), for a total of 64 sequencing libraries. The 16 samples used for this comparison came from a larger study investigating antimicrobial resistance and comprised composite fecal samples from pork, beef, poultry, and wastewater treatment plant (WWTP) operations (4 samples per source).

Our methodological objectives were to compare the results of each assay in order to evaluate the ability of the bait-capture and enrichment protocol to capture the low-abundance portion of the resistome, as well as virulence factors related to common enteric pathogens (see Additional file 1: Supplementary Materials and Methods), and to assess the use of UMIs for identifying and correcting amplification bias introduced by bait-capture and enrichment. We included enteric pathogen-associated virulence factors in our methodology due to their association with resistance evolution [25], as well as their importance in epidemiological outbreak investigations/surveillance [26, 27], evolution of resistant pathogens [28], and ongoing antibiotic resistance policy development [29]. Our overall objective was to characterize the low-abundance portion of the resistome and selected virulence factors (heretofore simply termed the “resistome”) as compared to the high-abundance portion and to determine whether this low-abundance portion could provide additional insight into resistome dynamics.

## Results

### Bait-capture and enrichment enabled access to > 1000 additional, low-abundance gene sequences

**Fig. 1:**
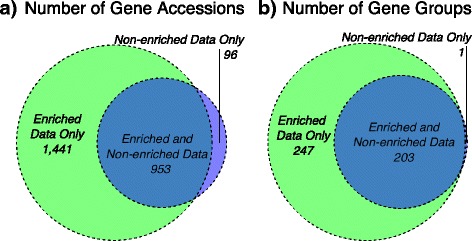
Venn diagram depicting the number of gene accessions (**a**) and gene groups (**b**) identified in enriched libraries only (green, *N* = 32 libraries); non-enriched libraries only (purple, *N* = 32 libraries); or both enriched and non-enriched libraries (overlapping blue areas, *N* = 64 libraries)

Across all 64 sequenced libraries, we identified 2490 unique antimicrobial resistance (AR), metal resistance (MR), biocide resistance (BR), and virulence factor (VF) gene accessions across 48 unique classes of resistance and virulence. Of the 2490 unique accessions, 2394 (96.1%) were identified in the sequencing data from samples subjected to capture and enrichment with MEGaRICH baits (i.e., Resistome and Resistome-UMI datasets, *n* = 32), 1049 (42.1%) were identified in non-enriched datasets (i.e., Metagenome and Metagenome-UMI datasets, *n* = 32), and 953 (38.3%) were common to both enriched and non-enriched datasets (*n* = 64). Therefore, using the custom MEGaRICH bait set, an additional 1441 unique gene accessions were identified compared to non-enrichment methods (Fig. 1a and Additional file 2: File S1). This represented more than a 100% increase in the number of unique accessions identified and therefore greatly expanded the detectable resistome. The majority of these additional 1441 genes originated from AR, BR, and MR genes (1155/1441 or 80.2%), and the majority of these were specific to AR (999/1155 or 86.5%, Additional file 2: File S1). A minority of the additional 1441 accessions were VF genes related to enteric pathogens (286/1441 or 19.8%). At the read level, the vast majority of the additional reads aligning to these 1441 genes originated from AR genes (87.3%), while 10.7% originated from VF genes and 2.0% originated from BR or MR genes. Cumulatively, reads aligning to these 1441 unique gene accessions accounted for just 1.8% of all reads that aligned to the resistance-virulence database across all 64 sequence sequencing libraries, indicating that these genes were part of the low-abundance or rare portion of the resistome.

These descriptive trends were reflected in statistical comparisons of gene richness between library preparation assays. Resistome-UMI libraries contained an average of 495 and 486 more unique gene accessions than the Metagenome-UMI and Metagenome libraries, respectively, while the Resistome libraries contained an average of 621 and 612 more unique gene accessions (Tukey *P* < 0.001 for all comparisons, Table 1). There were no differences in gene richness when comparing the Resistome versus Resistome-UMI and the Metagenome versus Metagenome-UMI libraries (Tukey *P* > 0.05). These results demonstrated that non-enriched shotgun metagenomic sequencing failed to identify hundreds of resistance gene accessions, even within a single sample.

**Table 1:** Diversity statistics, median (*range)*. Medians with different letters were significantly different (Tukey *P* < 0.05). Medians without letters were not subjected to pairwise comparison because the omnibus ANOVA test was not significant (*P* < 0.05)

Assay	Gene-level richness	Gene-level Simpson’s diversity	Gene-level Simpson’s equitability
Metagenome	277 (23–685)^ab^	14.5 (7.8–105.4)	0.14 (0.01–0.47)^abc^
Metagenome-UMI	366 (34–575)^ab^	23.8 (3.7–149.8)	0.11 (0.01–0.36)^ab^
Resistome	1074 (595–1330)^cd^	59.0 (9.2–142.5)	0.06 (0.01–0.11)^acd^
Resistome-UMI	766 (493–1265)^cd^	43.3 (16.5–144.4)	0.06 (0.03–0.12)^cd^

The type of assay also affected Simpson’s equitability, which is a measure of the evenness of gene distribution across the resistome. The Metagenome-UMI libraries had higher equitability than both the Resistome-UMI and Resistome libraries (Tukey *P* = 0.001 and 0.02, respectively), and the Metagenome libraries had higher equitability than the Resistome-UMI libraries (Tukey *P* = 0.008). There was no statistically significant difference in equitability for the comparison of the Resistome and Metagenome assays (*P* = 0.11) or when comparing Resistome-UMI versus Resistome and Metagenome-UMI versus Metagenome libraries (Tukey *P* = 0.99 for both comparisons). These results suggest that enrichment effectively expanded the resistome to include thousands of low-abundance genes, resulting in a less even distribution of abundance across the resistome. Interestingly, the type of library preparation assay did not exert a statistically significant effect on Simpson’s diversity index (ANOVA *P* = 0.06, Table 1), which is a measure of both the number of resistome elements within each sample (i.e., richness), as well as the abundance distribution of these elements (i.e., equitability). This suggests that the decreased equitability in the enriched samples compensated for the increased richness, resulting in no statistically significant differences in diversity.

### The low-abundance portion of the resistome differed significantly from the abundant portion

**Fig. 2:**
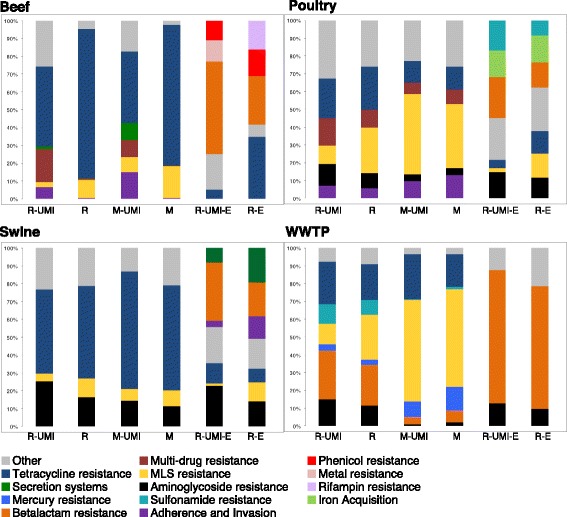
One hundred percent stacked graphs of resistome/virulome composition, by sample type and by library preparation assay (*R-UMI* Resistome-UMI, *R* Resistome, *M-UMI* Metagenome-UMI, *M* Metagenome, *E* composition in portion of the resistome identified only through enrichment). Proportional abundances were calculated by dividing the number of de-duplicated hits to each class by the total number of de-duplicated hits. Classes are shown individually if they contained at least 10% relative abundance in one of the assays within each sample type; all other classes were grouped into the “Other” category

Importantly, the additional 1441 identified gene accessions were not simply additional variants of resistance mechanisms that had already been identified in the non-enriched libraries. Rather, they comprised different proportions of resistance classes than the overall resistome (Fig. 2), indicating that the bait-capture and enrichment process were not simply detecting “more of the same.” At the most refined level of resistance classification (i.e., group, see Additional file 3: File S2), enrichment resulted in identification of an additional 247 AR, BR, and MR gene groups (Additional file 4: Table S1 and Fig. 1b). Of these additional 247 gene groups, 103 were found only in the enriched WWTP samples, 33 only in poultry, 11 only in beef, and 2 only in the enriched swine samples. These results suggest that the diversity in the low-abundance portion of the resistome may differ widely by sample type/source.

### The low-abundance portion of the resistome contained important and prevalent genes

**Fig. 3:**
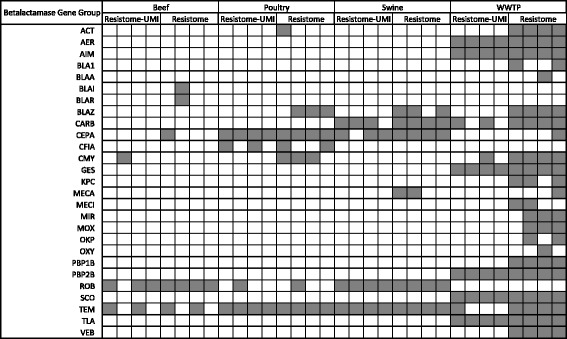
Binary heatmap of betalactamase groups identified only in enriched sequence libraries (*N* = 32). *Gray* present, *white* absent

The additional 247 gene groups identified with MEGaRICH included groups that confer resistance to antimicrobial drugs with high public health importance, such as extended-spectrum betalactamases (ESBLs) and carbapenemases CMY, KPC, TEM, GES, and VE-B types (Fig. 3). Interestingly, TEM betalactamases were identified in 29 of the 32 enriched libraries (i.e., Resistome and Resistome-UMI assays, Fig. 3), indicating that these genes were highly prevalent across the sample set, but present in very low abundance within each sample and therefore not detected without enrichment. The KPC ESBL group showed a similar pattern within WWTP samples (Fig. 3). These results highlight the insensitivity of non-enriched metagenomic sequencing for detecting high-importance, high-prevalence, but low-abundance genes that may be present in samples.

### The low-abundance resistome contained a higher diversity of resistance genes with highly informative SNPs

Use of MEGaRICH enabled identification of more read-pair haplotypes per gene (ANOVA *P* < 0.001), indicating that the low-abundance portion of the resistome contained more within-gene genetic diversity than the high-abundance resistome. Specifically, the Resistome dataset yielded an average of 1497 (range 259–3157) read-pair haplotypes per gene identified, while the Resistome-UMI, Metagenome and Metagenome-UMI datasets averaged 720 (range 44–2290), 57 (range 9–168), and 63 (range 11–143), respectively (Tukey *P* < 0.001 for all pairwise comparisons except between Metagenome and Metagenome-UMI, which was not significant).

**Fig. 4:**
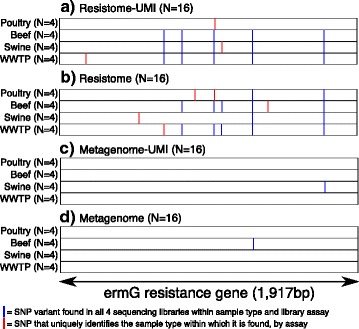
**a**–**d** Nucleotide variant depiction for the *ermG* gene (the length of which is represented on the *x*-axis) across all 64 samples (rows), grouped by library preparation assay (**a**–**d**) and sample type. Blue lines indicate SNPs identified in all 4 samples within sample type and assay, while red lines highlight SNPs that uniquely identify the sample type in which they are found by assay

Only one gene was identified in all 64 sequencing libraries, namely an *ermG* gene that mediates resistance to the macrolide-lincosamide-streptogramin class of antimicrobials [30]. We used this gene to illustrate the utility of comparing SNP patterns of AMR genes found in different samples, focusing only on SNPs that were found in all 4 samples from each sample type and each library assay. Within the Resistome and Resistome-UMI datasets, we identified unique *ermG* SNP patterns that could discriminate between sample type, i.e., beef, poultry, swine, and WWTP (Fig. 4a, b); this was not the case for the Metagenome and Metagenome-UMI datasets, presumably because of the fewer reads generated by shallower sequencing depth in the non-enriched samples (Fig. 4c, d).

### Bait-capture and enrichment did not impact overall resistome composition

**Fig. 5:**
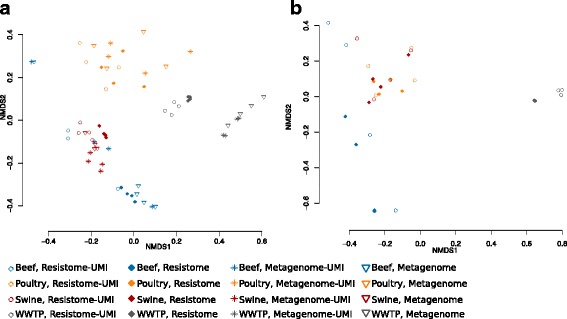
NMDS ordination of resistome composition at the class level, for **a** the entire resistome (i.e., all gene accessions identified) and **b** the low-abundance resistome (i.e., the additional gene accessions identified via enrichment). Each mark is one sample, mark color indicates sample type (*blue* beef, *orange* poultry, *red* swine, *gray* WWTP), and mark shape indicates library prep assay (*circle* Resistome-UMI, *diamond* Resistome, *asterisk* Metagenome-UMI, *triangle*, Metagenome). In both **a** and **b**, ordination by sample type was significant with a large effect size (*R* = 0.73 and *P* < 0.001 for both), while ordination by library preparation assay was significant but with a small effect size in **a** (*R* = 0.13 and *P* < 0.001) and insignificant in **b** (*R* = 0.54, *P* = 0.12). Three dimensions were used for ordination

Resistome composition at the class level differed greatly by type (i.e., beef, poultry, swine and WWTP), as indicated by ordination and the corresponding R-statistic (ANOSIM *R* = 0.73, *P* < 0.001, Fig. 5a), which measures the dissimilarity between groups; *R* values closer to 1.0 indicate high dissimilarity [31]. While the ANOSIM R-statistic for the dissimilarity between library preparation assays was statistically significant (*P* = 0.002), the *R* value was 0.13, suggesting that the type of library preparation assay did not account for large dissimilarities in resistome composition between samples. Within the WWTP and beef samples, there subjectively appeared to be separation by assay, but low sample numbers prevented us from performing ordination separately on these libraries (Fig. 5a). The influence of library preparation assay on resistome composition also was observed within specific classes of resistance (Fig. 2); for example, beef samples exhibited decreased relative abundance of tetracycline resistance genes in UMI libraries compared to non-UMI assays (Fig. 2). Ordination of samples based only on the low-abundance resistome (i.e., the 1441 gene accessions identified only via bait-capture and enrichment) continued to show significant separation by sample type (Fig. 5b, ANOSIM *R* = 0.73, *P* < 0.001), suggesting that the low-abundance portion of the resistome, while different in composition than the abundant portion, also differed significantly by sample type.

### Bait-capture and enrichment increased on-target sequencing without deficits in gene coverage

**Fig. 6:**
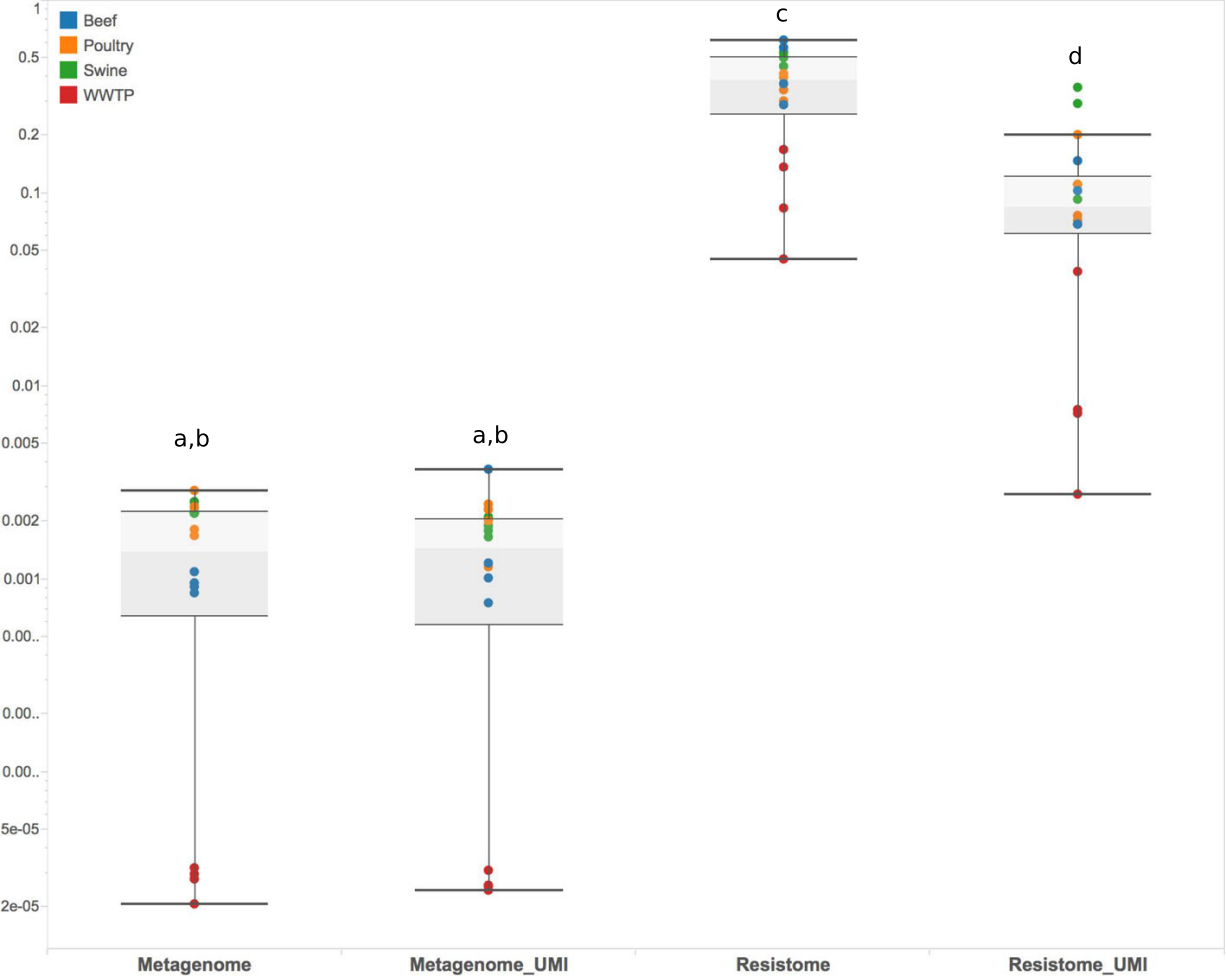
Boxplots of proportion of sequencing reads on-target (log-scale), by assay. Each dot is a sample (*n* = 64), colored by type (*blue* beef, *orange* poultry, *red* swine, *gray* WWTP). Boxes represent interquartile range and median, and whiskers represent range, except for outliers. Assays with different letters were significantly different (Tukey *P* < 0.05)

The proportion of on-target sequencing across all 64 sequencing libraries ranged from 0.002 to 61.8%, with statistically significant differences between all assays except Metagenome and Metagenome-UMI (Fig. 6). The use of MEGaRICH significantly increased on-target percentage from a median of 0.14% (range 0.002–0.37%) in the Metagenome and Metagenome-UMI datasets (*n* = 32) to 15.8% (range 0.28–68.2%) in the Resistome and Resistome-UMI datasets (*n* = 32). Within the latter sequencing libraries, the use of UMIs significantly decreased the on-target percentage (median 38.4% and range 4.5–61.8% for Resistome datasets versus median of 8.4% and range 0.28–35.4% for Resistome-UMI dataset). Across all 4 assays, WWTP samples tended to have a lower proportion of on-target reads compared to the other 3 sample types (Fig. 6). Bait-capture and enrichment resulted in positive log-fold change of database alignments across nearly all classes of resistance and virulence tested, with increases reaching statistical significance in 36 out of the 48 classes (Additional file 5: File S3).

The increase in on-target percentage in the Resistome and Resistome-UMI datasets was accompanied by an increase in gene coverage (i.e., proportion of nucleotides with at least 1 aligned read, Tukey *P* < 0.001, Table 2). In addition, evenness of coverage (as measured by Shannon Entropy and *L* ^2^-norm deviation per gene) did not differ between the Resistome dataset and the Metagenome and Metagenome-UMI datasets (Tukey *P* > 0.05), indicating that baits bound relatively evenly across the entire length of target genes when UMIs were not present. However, Resistome-UMI sequencing libraries exhibited higher Shannon entropy and *L* ^2^-norm deviation than sequencing data from the other 3 assays (Table 2, Tukey *P* < 0.001), indicating that UMIs produced less even coverage across genes.

**Table 2:** Target coverage statistics, median (*interquartile range)*. Medians with different letters were significantly different (Tukey *P* < 0.05). All models included sample type as a random effect

Assay	Gene coverage	Shannon entropy	*L* ^2^-norm deviation
Metagenome	0.63 (0.27–0.98)^ab^	0.91 (0.81–0.98)^abc^	0.987 (0.968–0.996)^abc^
Metagenome-UMI	0.57 (0.22–0.98)^ab^	0.90 (0.78–0.98)^abc^	0.985 (0.962–0.996)^abc^
Resistome	0.76 (0.32–0.99)^cd^	0.91 (0.81–0.96)^abc^	0.984 (0.966–0.992)^abc^
Resistome-UMI	0.83 (0.34–1.00)^cd^	0.94 (0.83–0.97)^d^	0.989 (0.971–1.000)^d^
Coefficients for sample type random effects	Beef 0.66 Poultry 0.76 Swine 0.75 WWTP 0.59	Beef 0.89 Poultry 0.91 Swine 0.91 WWTP 0.87	Beef 0.979 Poultry 0.983 Swine 0.983 WWTP 0.975

### Bait-capture introduced differing amounts of bias, which was corrected using UMIs

Through the de-duplication process (which used UMIs to correct for amplification bias), total numbers of unique reads within the Metagenome-UMI and Resistome-UMI sequencing libraries decreased from 742 to 738 M and from 760 to 508 M, respectively. Within the Metagenome-UMI dataset, the proportion of singletons (defined as UMIs with only one associated read-pair, i.e., without PCR duplicates in the sequence data) was > 99% (range 99.2–99.4%, Additional file 6: File S4). Within the Resistome-UMI dataset, an average of 45% of such reads was singletons, demonstrating that bait-capture and enrichment did indeed introduce amplification bias into the workflow. Amplification rates varied widely between sequencing libraries, ranging from 7.6–84.1%. The rates did not vary systematically with sequencing library diversity, richness, yield, or 260/280 ratio (Additional file 7: Figure S1) but did exhibit a logarithmic relationship with post-capture amplification DNA yield and proportion of post-capture DNA library used in sequencing (Additional file 8: Figure S2). The singleton rate for each sequencing library seemed to dictate the singleton rate for all genes identified within the library, as rank-abundance curves showed a lack of positive or negative relationship (Additional file 9: Figure S3).

## Discussion

The limits of sequencing technology to detect low-abundance members of ecosystems have been recognized for many years [32]. Here, we demonstrated the existence, extent, and importance of this detection limit for resistome analyses, by the use of shotgun metagenomic sequencing of non-enriched and enriched DNA from the same samples, using the same analytical methods. Our results showed that traditional (i.e., non-enriched) shotgun metagenomic sequencing failed to detect the low-abundance members of the resistome, which comprised 247 additional gene groups specific to antimicrobial resistance, represented by 1155 additional resistance-related gene accessions. These additional gene groups included ESBLs and carbapenemases, which are important from a clinical and public health standpoint (Fig. 3 and Additional file 4: Table S1). Although livestock resistomes have not been heavily studied, available data corroborate the sparsity of these resistance genes within livestock-associated samples sequenced using non-enriched metagenomic methods [11, 33–35], with the exception of bla-TEM genes detected in livestock fecal samples collected in China [36]. Increased sensitivity for rare yet clinically important sequences is especially important given interest from the scientific and regulatory communities in metagenomic sequencing for pathogen detection and AMR surveillance [37–39]. However, it should be noted that the risk of low-abundance AMR genes within metagenomic samples is not yet well understood [40]. Genomic context, i.e., location on a mobile genetic element or within the genome of a pathogenic bacterium, is a critical piece of information in assessing such risk. Currently, metagenomic data are not conducive to genomic localization, as the assemblies tend to be highly fragmented [41]. Therefore, until the risk of these low-abundance genes is better understood, large-scale surveillance efforts that utilize bait enrichment and/or metagenomic sequencing should include follow-up isolation and/or PCR of identified genes to better assess their clinical and/or public health significance. Similarly, studies wishing to investigate the effects of selection pressure on evolution of low-abundance genes within the context of the resistome should incorporate external methods of measuring gene abundance, at least until the correlation between these more traditional methods and metagenomic quantification is better understood [42].

In addition to describing the diversity of the low-abundance resistome, we also demonstrated that it contained a high level of within-gene variation. We used this variation to successfully discriminate between sample types, which was not possible in the non-enriched datasets (Fig. 4). Such genetic information could be very powerful for tracking and attribution of resistance genes within environmental and clinical metagenomics samples. This approach has been demonstrated with whole-genome sequence data from isolates obtained during several outbreak scenarios [43, 44]. Follow-up study with more numerous and representative samples is needed to understand both the full potential and limitations of metagenomic data within this context.

The additional genes identified through bait-capture and enrichment did not appreciably alter resistome composition (Fig. 5), despite the fact that the composition of these additional genes differed from the total resistome (Fig. 2). This dynamic likely resulted from the relatively low abundance of these additional genes in the overall resistome (< 2% of all aligned reads), as well as the fact that resistome composition was driven primarily by differences in sample source (i.e., beef, poultry, swine, or WWTP, Fig. 5). This latter finding could be due to major microbiome differences between the sample types (Additional file 10: File S5), although there are many potentially confounding factors including geographic location, host species, and antibiotic usage patterns. Regardless of the factors that may have impacted differences between resistomes, the results obtained via enrichment suggest that the distribution of genes within resistomes exhibited a “long tail” comprised of many unique, yet rare genes, as has been described for the human microbiome and ecological dynamics across systems [14, 45, 46]. The limitations of NGS to detect the “rare biosphere” could constrain inferences about ecological dynamics and diversity [46]. Rarefaction curves for the 64 libraries in this study provided further evidence of this “long tail,” as the curves increased steeply and quickly before beginning a very long, gradual yet steady incline with increasing sequencing depth (Additional file 11: Figure S4). Even with enrichment, these curves never completely leveled off (although the rate of detection of new genes slowed considerably), suggesting that the complete sequencing diversity of these samples has not been completely characterized.

Currently, there are few methods available for enriching the rare resistome within metagenomic samples. Functional metagenomics is one such method that enriches AMR genes by constructing cloned metagenomic libraries that are then exposed to antibiotic-impregnated culture media; however, this approach necessitates laborious and expensive lab-based techniques, which render the approach unsuitable for high-throughput applications such as ongoing, large-scale AMR surveillance programs [47]. The bait-capture and enrichment assay utilized in this work followed a relatively simple protocol and achieved a 2–4 order of magnitude increase in on-target sequence data (Fig. 6 and Additional file 5: File S3). The MEGaRICH bait set is publicly available (Additional file 12: File S6) and can be easily updated to incorporate additional target sequences.

Many resistome projects aim to characterize and/or compare the resistome within and across samples, which requires the ability to accurately quantify DNA sequences. This, in turn, relies on the random nature of library preparation and sequencing, as well as the assumption that each piece of DNA is represented by a single sequenced read. Traditional shotgun metagenomics typically meets this assumption in samples with ample bacteria because the amount of DNA is so large that even DNA from the most abundant species or genes will only be sequenced once. We verified this fact using UMIs in non-enriched samples, which showed that > 99.9% of sequence reads were singletons (i.e., sequenced only once). However, our use of UMIs also demonstrated that bait-capture and enrichment differentially amplified DNA across samples. We hypothesized that this was driven largely by the target-to-bait ratio and that samples with a more abundant resistome (and thus more targets) would yield a higher proportion of singletons. However, if this were true, we would expect the relatively small resistome in the WWTP samples to exhibit much lower singleton rates than libraries from the other three sample types (Fig. 6). We did not find this to be the case (Additional files 7, 8, and 9: Figures S1–S3), and we can only posit that the target-to-bait ratio is dictated by a complex interplay of resistome composition, degree of matching between targets and baits, and DNA quality within each sample.

Given the differential amplification bias induced though bait-capture and enrichment, concurrent use of UMIs (or some other method for de-duplicating sequence data) is necessary if the goal of analysis is to quantify or compare resistome composition within or between samples. The use of UMIs, however, came at the cost of decreased on-target sequencing (Fig. 6), perhaps because our protocol did not include “blockers” for UMI sequences. In standard bait-capture protocols, blockers increase capture efficacy by masking adapters so that baits do not bind to off-target sequence. The fact that UMIs also decreased evenness of coverage (Table 2) suggests that UMIs may have impeded bait binding within targets. Future optimizations of our pre-sequencing capture design should attempt to incorporate UMI blockers to mitigate depression in on-target sequencing. Similarly, future work should attempt to standardize the enrichment and UMI protocols around an optimal insert size as well as a consistent sequencing depth. The insert sizes and total sequence reads in this work were significantly different between assays (Table 3) because standard kit shearing protocols were followed, and these protocols differed between the enrichment and library preparation kits (Additional file 1: Supplementary Materials and Methods). While we attempted to control for this difference by including insert size and total sequence reads as confounders in mixed-effect models (see Additional file 1: Supplementary Materials and Methods), it is unknown whether or how such differences may ultimately have impacted the ability to enrich for, sequence, and then computationally identify targets [48, 49].

**Table 3:** Sequencing statistics, median (*range)*. Medians with different letters were significantly different (Tukey *P* < 0.05). All models included sample type as a random effect

Assay	Raw reads (M)	Trimmed, non-host reads (M)	Insert size	Phred score
Metagenome	39.9 (14.2–55.3)^a^	38.8 (14.1–53.4)^a^	346 (322–380)^a^	35.0 (34.6–36.1)^ac^
Metagenome-UMI	43.5 (33.3–65.4)^bd^	42.0 (31.0–62.7)^bd^	315 (301–343)^bd^	34.8 (34.2–35.0)^bd^
Resistome	65.4 (54.7–79.1)^c^	64.0 (53.3–77.7)^c^	179 (146–186)^c^	35.1 (35.0–35.4)^ac^
Resistome-UMI	49.1 (27.6–57.7)^bd^	46.9 (24.5–55.2)^bd^	328 (315–353)^bd^	34.6 (33.9–35.0)^bd^

There are several important limitations to the overall approach described in this report. First, baits were designed based on known gene sequences and thus were not able to capture completely novel AR, BR, MR, and VF genes. While binding affinity tolerates approximately 40 mismatches between bait and target (and therefore allows for sequence variant detection), even this level of matching prevents novel gene discovery, which can be accomplished with functional metagenomic screening [50]. Second, reliance on reference databases likely introduced bias into the bait set design, as some resistance and virulence classes were overrepresented in the literature. While the use of a mock metagenomic community could help to correct for such bias, there are logistical challenges to such an approach, including the ability to isolate and clone all of the AR, MR, BR, and VF gene accessions that were included in the MEGaRICH bait set.

Overall, our results suggest that the low-abundance portion of the resistome contains additional and valuable information that could substantially alter our understanding of resistome dynamics, while improving our ability to track resistance genes across metagenomic samples. However, we have demonstrated that standard metagenomic sequencing approaches do not capture the low-abundance portion of the resistome. MEGaRICH, a bait-capture and enrichment system, greatly increased our ability to detect the low-abundance portion of the resistome, but also introduced amplification bias into the data. This was corrected with UMIs but at the cost of decreased amplification efficiency. Therefore, choice of enrichment assay should ultimately be guided by the primary study objective. If the goal is presence/absence of rare resistome elements (including rare SNPs), then pre-sequencing capture without UMIs would be preferable. However, if quantification of the resistome is desired, UMIs should be incorporated into the workflow.

## Conclusions

Antimicrobial resistance (AMR) has been recognized as a critical threat to public health. AMR is a complex phenomenon mediated by interactions between microbes, their hosts, and surrounding environmental conditions. Historically, our ability to investigate the complex interactions underlying AMR has been limited by technological constraints on our access to the microbial community. The present study uses a technique to enrich resistance sequences within metagenomic samples, resulting in increased detection of AMR and discovery of a rare resistome with features that are distinct from the more abundant portion of the resistome. These distinct features not only expand the utility of resistome analysis but also modify our understanding of AMR ecology within the context of microbial communities. Furthermore, the discovery of AMR genes with high public health importance within the rare resistome highlights the need for increased sensitivity of metagenomic methods, particularly when they are applied to public health surveillance. Therefore, the methods presented in this study should find widespread utility within both microbiome-resistome research and more practical applications of metagenomic sequencing. Finally, our results suggest that detection of rare resistance elements is critical for advancing efforts to combat AMR.

## Methods

### Design of capture baits used in the MEGaRICH system

Publicly available databases were used for design of a custom set of 120-mer biotinylated cRNA baits that form the foundation of the MEGaRICH system. Bait design was based on a non-redundant list of gene nucleotide sequences for all known antimicrobial resistance (AR), metal resistance (MR), biocide resistance (BR), and virulence factor (VF) genes. See Additional file 1: Supplementary Material and Methods for details on the bait design process. Briefly, AR gene sequences were compiled from Resfinder [51], ARG-ANNOT [52], CARD [53], and the Lahey Clinic betalactamase database [54]. Metal and biocide resistance sequences were collected from the BacMet database [55]. Virulence factor sequences specific to the pathogens *Escherichia coli*, *Enterococcus* spp., and *Salmonella* spp. were compiled from revisions one [56] and three [57] of the Virulence Factor Database. The final non-redundant list contained 5557 gene accessions (complete database available at http://hdl.handle.net/10217/180280). Optimization allowed us to condense the amount of target sequence from 6.98 to 3.34 Mb so that the final MEGaRICH bait set comprised 31,250 unique 120-mers (Additional file 12: File S6), which fit easily within the smallest and least expensive tier size (i.e., < 499 kbp) for Agilent’s SureSelect^XT^ Custom Capture Library (Agilent Technologies, ELID number 0792071).

### Library preparation, target capture/enrichment, and UMIs

DNA was extracted from samples using the PowerMax Soil DNA Isolation Kit according to manufacturer’s instructions, with minor modifications (see Additional file 1: Supplementary Methods). Library preparation of Metagenome and Resistome libraries followed standard commercial kit protocols, with some modifications. For the Metagenome libraries, the TruSeq DNA PCR-Free LT Library Prep Kit (Illumina) was used, while resistome libraries were created using the SureSelect^XT^ Target Enrichment System for Illumina Paired-End Multiplexed Sequencing Library (Agilent Technologies) with the custom-designed MEGaRICH bait set. The Resistome-UMI and Metagenome-UMI libraries were created using a protocol that incorporated dual-indexed UMI adapters (see Additional file 13) into sequence libraries [23], with modifications to allow for integration with Agilent SureSelect protocols (see Additional file 1: Supplementary Methods for details).

### Sequencing

All 64 sequencing libraries were sequenced at a depth of four libraries per lane (i.e., 16 lanes total) on a HiSeq 2500 (Illumina) with 2 × 125 bp paired-end reads using HiSeq SBS Kit v4 reagents (Illumina). Fastq files for all sequencing runs are available on NCBI SRA under Project PRJNA339554. A total of 3.13B paired-end reads were produced. Total raw reads per sequencing library were higher in the Resistome-UMI and Metagenome-UMI datasets compared to the Metagenome dataset (Tukey *P* < 0.05, Table 3; for complete sequencing statistics, see Additional file 6: File S4) and therefore were included as a potential confounder in all analyses of capture efficiency (see Additional file 1: Supplementary Methods). The average quality score and insert size also differed by assay, with the UMI datasets exhibiting slightly lower Phred scores (Tukey *P* < 0.05, Table 3) and the Resistome and Metagenome datasets containing smaller and larger insert sizes than the other assays, respectively (Tukey *P* < 0.05, Table 3). Differences in insert size resulted from differences in library preparation assays, i.e., the standard bait enrichment process used to create the Resistome libraries included a longer shearing time, resulting in smaller insert sizes. Conversely, Metagenome libraries were created using the standard TruSeq DNA PCR-Free LT Library Prep Kit (Illumina), which resulted in a longer insert size. The UMI libraries necessitated use of a custom protocol, which ended up generating insert sizes in-between those for the Resistome and Metagenome libraries (see Additional file 1: Supplementary Materials and Methods for details on all library preparation procedures).

### Bioinformatics analysis

In order to identify and remove read duplicates within the UMI libraries, the tag_to_header.py script published in [23] was used along with a custom C++ program (https://github.com/cdeanj/meg_scripts/tree/master/umi). See Additional file 1: Supplemental Methods for details. De-duplicated sequence reads were trimmed and filtered, and then host-associated sequence reads were identified and removed. Across all 4 datasets, a median of 3.3% of reads were removed due to low quality (range 1–11%), and a median of 0.07% of high-quality reads were identified as host DNA and removed from further analysis (range 0.0009–3.1%, see Additional file 6: File S4).

Because a major update had been made to one of the source databases used for bait design, an updated AR nucleotide database was used to identify AR, MR, BR, and VF genes within the sequence data (see Additional file 1: Supplementary Methods for details, and http://hdl.handle.net/10217/180280 for the updated version of the database). The addition of new gene sequences in this database also allowed us to test the ability of MEGaRICH to identify sequence variants not included in the original dataset. Trimmed, non-host reads were aligned to this database, and genes with at least 80% gene fraction were included in further analyses (see Additional file 1: Supplementary Methods for details). Genes were classified by resistance/virulence class (e.g., tetracycline or betalactam drug classes) and gene group (e.g., tet-Q or betalactamase-TEM) in order to further characterize the resistome of the sample.

In order to identify and count sequence variants within AR, MR, BR, and VG genes, SNPFinder [58] was used to identify one or more single-nucleotide polymorphisms (SNPs) within aligned reads and concatenate them into a read-pair haplotype, i.e., a pattern of SNPs within a sequence read-pair. The average number of unique read-pair haplotypes identified per gene within a sequencing library was compared in order to evaluate how effectively the different assays detected sequence variability. Genes identified in all 64 sequencing libraries were subjected to further SNP analysis to assess variant stratification by sample type and library assay (see Additional file 1: Supplementary Method for details).

### Descriptive and statistical analyses

A number of metrics were used to evaluate potential differences between library preparation assays (see Additional file 1: Supplementary Methods for details). To assess potential bias introduced by sequencing depth or quality, we compared the numbers of raw, filtered and host reads; median insert size; and average quality score. To quantify the efficacy of the enrichment process, we compared both the proportion of on-target sequence reads (i.e., the proportion of reads that aligned to the resistance/virulence database) and the log-fold change in alignments per resistance/virulence class. To evaluate the ability of the assays to provide complete and uniform coverage across targets, we compared the depth, breadth, and evenness of coverage (the latter of which was measured using Shannon Entropy and *L* ^2^-norm deviation). Linear mixed models were used to assess statistical significance of all of these comparisons. To compare overall resistome composition across assays, ordination using non-metric multidimensional scaling (NMDS) was performed. Scatterplots were used to analyze trends between amplification bias (measured using the proportion of non-amplified DNA sequences) and sample-level characteristics including number of raw reads, DNA quality, and resistome diversity and richness.

## Additional files

Additional file 1: Supplementary materials and methods (DOCX 59 kb)

Additional file 2: File S1. Additional gene accessions identified using bait enrichment. (XLSX 79 kb)

Additional file 3: File S2. Classification of genes at the class, mechanism and group levels. (CSV 764 kb)

Additional file 4: Table S1. Number of aligned reads to each gene group identified only in enriched samples, by resistance class and sample type. (DOCX 41 kb)

Additional file 5: File S3. Log-fold change in abundance for each class of resistance, comparing enriched versus non-enriched libraries. (XLSX 31 kb)

Additional file 6: File S4. Per library sequencing and alignment statistics. (XLSX 51 kb)

Additional file 7: Figure S1. Proportion of non-PCR amplified reads within the Resistome-UMI dataset as a linear function of (a) resistome richness at the gene level (*R* ^2^ = 0.10), (b) Simpsons diversity at the gene level (*R* ^2^ = 0.26), (c) total number of raw sequence reads (*R* ^2^ = 0.31), and (d) average NanoDrop value (*R* ^2^ = 0.006). Each dot is a sequencing library (*N* = 16); blue = beef, orange = poultry, red = swine, grayWWTP. (EPS 148 kb)

Additional file 8: Figure S2. Proportion of non-PCR amplified reads within the Resistome-UMI dataset as a function of (a) capture efficiency and (b) proportion of DNA library pooled for sequencing. Each dot is a sequencing library (*n* = 16); blue = beef, orange = poultry, red = swine, gray = WWTP. Logarithmic trend lines with 95% confidence intervals depicted in dashed lines (*R* ^2^ = 0.93 and 0.98, respectively). (EPS 180 kb)

Additional file 9: Figure S3. Proportion of non-amplified PCR reads within each gene (*y*-axes) as a function of gene rank abundance (*x*-axes) within the Resistome-UMI samples (*n* = 16), separated by samples obtained from (a) beef, (b) poultry, (c) swine, and (d) WWTP facilities. Each color within each panel is a single sequencing library, and each dot is an AR, BR, MR, or VF gene within that library. (EPS 399 kb)

Additional file 10: File S5. Matrix of species-level counts, by sample. (XLSX 761 kb)

Additional file 11: Figure S4. Rarefaction curves for (a) samples subjected to pre-sequencing enrichment and (b) subjected to non-enriched shotgun metagenomic library preparation. Blue, beef; orange, poultry; red, swine; gray, WWTP. Solid lines are samples with UMIs, and dashed lines are samples without UMIs. (EPS 92 kb)

Additional file 12:. File S6. All of the 120-mer bait sequences used for the MEGaRICH bait capture and enrichment assay, including target gene accessions. (XLSX 2035 kb)

Additional file 13: Table S2. Primers used in custom dual-indexed UMI protocol. (DOCX 68 kb)

## Abbreviations

AMR: Antimicrobial resistance
ANOSIM: Analysis of similarities
ANOVA: Analysis of variance
AR: Antibiotic resistance gene accession
BR: Biocide resistance gene accession
DNA: Deoxyribonucleic acid
ESBL: Extended-spectrum betalactamase
MR: Metal resistance gene accession
NMDS: Non-metric multidimensional scaling
SNP: Single-nucleotide polymorphism
UMI: Unique molecular index
VF: Virulence factor gene accession
WWTP: Wastewater treatment plant
