# Supplementary material for: Enrichment allows identification of diverse, rare elements in metagenomic resistome-virulome sequencing
Source: Microbiome. 2017 Oct 17;5:142. doi: 10.1186/s40168-017-0361-8 (PMC5645900; doi:10.1186/s40168-017-0361-8)
Supplement: Supplementary file 1 — Supplementary materials and methods (DOCX 59 kb) [file 40168_2017_361_MOESM1_ESM.docx]

**Supplementary Methods**

*DNA Extraction Details*

Selected samples were thawed at room temperature, and a 10g aliquot from each was placed in a 50ml conical for total DNA extraction using the PowerMax Soil DNA Isolation Kit (MO BIO Laboratories). For all raw fecal samples (beef, pork, poultry), a pre-sedimentation step was conducted to remove fecal debris and inhibitors. To each 10g sample, 30 ml buffered peptone water (BPW) was added and the conical was shaken until well mixed. Large particles were allowed to settle out of suspension for 5-10 minutes, and the supernatant was removed to a clean conical. Conicals were centrifuged at 4300 x g for 10 min, the supernatant poured off, and the resulting pellet resuspended in 5ml of 1X phosphate-buffered saline (PBS). Centrifugation and removal of supernatant were repeated. DNA was extracted from the pellet or 10g of non-sedimented sample (WTTP samples) according to manufacturer’s instructions, with a final DNA elution into 3 ml C6 buffer. DNA was concentrated by a standard sodium acetate/ethanol precipitation procedure and resuspended in 150 µl C6 buffer. DNA concentration was assessed using the Qubit dsDNA HS Assay Kit on a Qubit 2.0 Fluorometer (Thermo Fisher Scientific). DNA quality (260/280 ratio) was assessed using a NanoDrop ND-1000 (Thermo Fisher Scientific).

*SureSelect Protocol Details for Resistome Library*

To obtain DNA fragments around 200-300bp, genomic DNA (3µg DNA in 130 µl 1X low TE buffer) was sheared on an M220 Focused-ultrasonicator (Covaris) in microTUBE AFA Fiber Pre-Slit Snap-Cap 6x16mm tubes (Covaris) at the following settings: 10% duty factor, 50 peak incident power, 200 cycles/burst, 6°C, 240s. Subsequently, the remainder of the SureSelect^XT^ protocol for 3 µg DNA samples was followed, with a few modifications. Pre-capture PCR amplification was conducted using all of the adaptor-ligated library in order to maintain library diversity, using 4 amplification cycles. Post-capture PCR was conducted using all of the library-bound beads, using 15 amplification cycles.

*Details for Creation of UMI Libraries*

UMI adapters were used in conjunction with reagents from the SureSelect^XT^ Fast Target Enrichment System for Illumina Paired-End Multiplexed Sequencing Library (Agilent Technologies). UMI adapters were synthesized using the adapter oligos MWS51_KJ and MWS55_KJ (Table S2) according to the previously published “Synthesis of DS adapters” protocol(Kennedy *et al.*, 2014).

Metagenome-UMI libraries were created using the SureSelect^XT^ protocol, following the library prep part of the protocol for 3 µg DNA samples, with modifications. To obtain DNA fragments around 450-550bp, genomic DNA was sheared as stated previously, except the time was decreased to 210s. Two shear volumes per sample (4µg DNA in 130 µl 1X low TE buffer) were processed for a total of 8µg. Agencourt AMPure XP PCR Purification paramagnetic beads (Beckman Coulter) were used for size selection to achieve the desired fragment length by adding a ratio of 0.65 beads:sample volume. For library prep, 1400-1600 ng of sheared, size-selected DNA was added to the end repair step. After the A-tailing step, the DNA concentration was measured by Qubit, and 1000-1400 ng A-tailed DNA was used for ligation. Instead of the SureSelect Adapter Oligo Mix, 0.3 µl UMI adapters were added per 250 ng A-Tailed DNA. To amplify the UMI adapter-ligated library, SureSelect primers in the Pre-Capture PCR Reaction Mix were replaced with 10µM i7 and i5 Index Primers (2 µl each, Table S2). Unique Index Primers were created using one of the 8 bp indices listed in the SureSelect^XT^ protocol reference section.

Resistome-UMI libraries were created using the SureSelect^XT^ Fast protocol, following the Hybridization and Capture part of the protocol, with modifications. For Hybridization, 500-1000ng of each Metagenome-UMI library was used. The Fast Hybridization protocol was followed; however, the final 65°C hold step was extended to 16 hrs (to match the hybridization time in the standard SureSelect^XT^ protocol), prior to completing the post-hybridization capture steps. The post-capture PCR was conducted for 13 cycles using the primers MWS13 and BP2 (Kennedy *et al.*, 2014) (Table S2) at 0.4 µM each instead of the SureSelect PCR Primer Mix.

Prior to sequencing, Metagenome-UMI and Resistome-UMI libraries were size-selected using a BluePippin (Sage Science). For each type of library prep, the 16 libraries were pooled in amounts equimolar for the region to be selected (400-800bp). Library pools were then size-selected for fragments from 400-800bp using a BluePippin DNA Gel Cassette, 1.5% agarose, 250bp-1.5kb (Sage Science).

*Details on Creation of Non-Redundant List of Capture Baits*

Redundant sequences between ARG-ANNOT (Gupta *et al.*, 2014) and Resfinder (Zankari *et al.*, 2012) - both downloaded on August 12^th^, 2014 - were identified using CD-HIT-EST-2D (Fu *et al.*, 2012) with local alignment (‑G 0) and the following parameters: -c 1.0 -AS 0 -AL 0 -aL 1.0 -aS 1.0. A single representative sequence was selected from each resulting cluster (n=1,427), and appended to the list of unique gene sequences in ARG-ANNOT (n=261) and Resfinder (n=715). This process was then repeated for the CARD database (McArthur *et al.*, 2013) (also downloaded on August 12^th^, 2014) using the combined ARG-ANNOT/Resfinder non-redundant database. Seven hundred and eight sequences were unique to CARD, resulting in a final non-redundant list containing 3,111 unique AR gene sequences. Next, revisions one (R1) (Chen *et al.*, 2005) and three (R3) (Chen *et al.*, 2012) of the Virulence Factor Database were downloaded (March 4^th^, 2015), and any accessions associated with the pathogens *Escherichia coli*, *Enterococcus* spp. and *Salmonella* spp. were extracted and similarly collapsed using CD-HIT (n=432). Accessions encoding for metal and biocide resistance were downloaded as amino acid sequences from the BacMet database (Pal *et al.*, 2014) of experimentally confirmed genes (April 6^th^, 2015). In order to convert these into nucleotide sequences, each accession was submitted to UniProt’s “Cross-references” section, which is maintained by the National Center for Biotechnology Information (NCBI) and the European Bioinformatics Institute and is able to link protein-level accessions to corresponding nucleotide accessions. In all, 1,642 nucleotide sequences were identified, and redundant sequences were removed using CD-HIT-EST as described above, resulting in 862 MR and BR genes. Finally, complete sequences from the Lahey Clinic betalactamase database (Bush and Jacoby, 2010) (n=1,153 including plasmids) were added for a combined list of 5,557 AR, BR, MR and VF gene accessions (available at http://hdl.handle.net/10217/180280).

The list of 5,557 gene accessions was further optimized in order to maximize efficiency of the capture design. This optimization process was undertaken based on parameters supplied by Agilent. First, all sequences were fragmented *in silico* into consecutive 120-mers, the length of an Agilent SureSelect biotinylated cRNA bait; for fragments at the end of genes, the start position was left-shifted until a full 120-mer was achieved. All 120-mers were then clustered using CD-HIT (Fu *et al.*, 2012); a conservative clustering similarity threshold of 80% was chosen based on information from Agilent that the SureSelect System is designed to tolerate up to 40 mismatching bases within a 120-mer. A single representative 120-mer was then selected from each CD-HIT cluster, and these 120-mers were then matched back to the sequences from the original list using BLAT (Kent, 2002) to ensure that all AR, MR, BR and VF genes would be captured by at least one non-redundant 120-mer bait. Genes that were not captured were tiled at 1x coverage and the resulting 120-mer sequences were added manually to the list of 120-mers for eventual production. This process produced a list of 27,841 unique 120-mers, which were sent to Agilent for manufacture. Agilent then applied a custom algorithm that creates *in silico* copies of high-GC content 120-mers (“balanced boosting”); this process can result in a maximum of 16 copies of a single 120-mer. The final bait set thus contained 31,250 baits (File S6).

*Details for Bioinformatic Analysis*

After using the tag_to_header.py script from (Kennedy *et al.*, 2014), a custom C++ program (<https://github.com/cdeanj/meg_scripts/tree/master/umi)> was used to identify and collapse UMI families (i.e., reads with the same 24-mer UMI sequence). To avoid making any assumptions about the “true” sequence, a N-substitution was made for any sequence mismatch at any frequency. After the N-substitution process was complete, a single N-replaced representative sequence was then chosen for further analysis (i.e., “de-duplication”). When no such variability existed within a UMI family (i.e., all of the members of the family had the exact same sequence), a single representative was chosen for further analysis. For both N-replaced and exact-match representative sequences, the read ID was appended with the number of reads originally represented within that UMI family, and the Phred scores for the last representative in the family were chosen for use in downstream analyses.

Trimmomatic (Bolger *et al.*, 2014) was used for read trimming and filtering, using the following settings: ILLUMINACLIP:”TruSeq3-PE.fa:2:30:10:3:TRUE" LEADING:3 TRAILING:3 SLIDINGWINDOW:4:15 MINLEN:36.

After trimming and filtering, BWA (Li and Durbin, 2009) was used to align reads to host genomes as follows: *Gallus gallus* (NC_006088.3) for poultry facility samples, *Sus scrofa* (NC_010452.3) for swine facility samples, *Bos taurus* (AC_000158.1) for beef facility samples, and *Homo sapiens* (NC_000001.11) for human WWTP facility samples. Reads that aligned to these genomes were then removed from further analysis using samtools (Li *et al.*, 2009).

Trimmed, filtered and nonhost reads were then aligned to a reference database of AR, MR, BR and VF gene accessions. Because a major update had been made to one of the source databases used to create the baits (i.e., CARD) (McArthur *et al.*, 2013), an updated AR nucleotide database was used to identify AR, MR, BR and VF genes within the sequence data. The core AR database content was obtained by non-redundant inclusion of sequences contained in Resfinder as accessed in November 2015 (Zankari *et al.*, 2012), ARG-ANNOT as accessed in November 2015 (Gupta *et al.*, 2014), CARD v1.0.7 (McArthur *et al.*, 2013), and NCBI Lahey Clinic beta-lactamase archive as accessed in December 2015 (Bush and Jacoby, 2010). All data were included except for the CARD protein wild-type sequences, as these represented genes that do not carry a known resistance mutation. Before inspection, sequences were collapsed at 100% identity using BLAT (v36x1) (Kent, 2002) with a maximum gap allowance of 0 to produce a set of unique sequences. For entries that contained header information with an NCBI accession number, Coding Sequence (CDS) regions were obtained by querying NCBI using the BioPython (v1.66) (Cock *et al.*, 2009) module and the NCBI Entrez eUtils interface. Entries with multiple CDS were split into separate sequences along CDS boundaries and re-annotated based on the NCBI CDS annotation. Gene annotations were validated through a combination of translated BLAST at > 90% identity and gene clustering with USEARCH (Edgar, 2010) at > 80% identity. Genes that were identified as not related to AMR were excluded from the database. The resulting database contained 3,824 AR genes (Lakin *et al.*, 2016), which were combined with MR, BR and VF genes using CD-HIT-EST (Fu *et al.*, 2012) for a final alignment database containing 11,157 unique accessions (available at http://hdl.handle.net/10217/180280).

Gene fraction was defined as the proportion of nucleotides within the reference sequence that were aligned by at least 1 sequence read. This cutoff was designed to decrease the number of false positive gene identifications within the metagenomic data (Gibson *et al.*, 2015). Genes and reads that did not make this cutoff were not included in downstream statistical analyses.

Freebayes (Garrison and Marth, 2012) was used to call sequence variants on genes that were present in all 64 sequencing libraries. Freebayes was run assuming a pooled sample with unknown numbers of organisms and a ploidy of 1. A minimum coverage value of 5 and a frequency threshold of 0.01 were used for variant calling.

Trimmed, filtered, nonhost reads were also subjected to microbiome analysis using kraken with default settings (Wood and Salzberg, 2014). The kraken database was modified based on the recommendation of kraken’s developers (see full script at (<https://github.com/colostatemeg/meglab-kraken-custom-db)>, i.e., with removal of non-taxonomic plasmid sequences and masking of low-complexity regions (Morgulis *et al.*, 2006). Counts were aggregated to the species level and a raw count matrix of reads per species by sample was output.

*Descriptive and Statistical Analysis*

In order to appropriately compare the proportion of raw, trimmed, filtered and on-target reads among the 4 library preparation assays, we needed to re-inflate the read counts within UMI families. This was done using a custom C++ program (https://github.com/cdeanj/meg_scripts/tree/master/umi_count) that output a histogram of UMI family sizes for each read within the dataset, i.e., within trimmed and filtered fastq files and within SAM alignment files. When comparing resistome composition between samples, non-reinflated (i.e., deduplicated) read counts were used in order to correct for duplication bias.

To assess potential bias by sequencing depth or quality, numbers of raw, filtered and host reads, as well as median insert size and average quality score, were compared among assays using linear mixed models with sample type (i.e., beef, poultry, swine or WWTP) and sample ID (i.e., repeated measurements per each of the 16 samples) included as a random-intercept and random-slope effect (Smyth, 2004). Values for raw, filtered and host reads were scaled for model stability. The significance of the library preparation assay on these metrics was tested using a log-likelihood test comparing nested models with and without assay as a predictor variable, and Tukey all-pair contrasts between the 4 assays were conducted using generalized linear hypothesis tests to correct for multiple comparisons of means (Hothorn *et al.*, 2008).

In order to assess efficacy of each library preparation assay in targeting AR, MR, BR and VF genes, compared the proportion of on-target sequence reads between assay, which was defined as the sum of reads in a sample aligning to a gene in the database divided by the total number of raw reads in the same sequencing dataset, using mixed-effects models as described above. For data with UMIs, reinflated counts were used for the total of both the aligned and raw reads. We also compared the log-fold change per resistance/virulence class between assays using zero-inflated Gaussian mixture models of counts transformed using cumulative sum scaling to account for uneven sequencing depth, with sample type added as a fixed effect to control for potential confounding (Paulson *et al.*, 2013). Pairwise comparisons of log-fold change in abundance between enriched libraries (i.e., Resistome and Resistome-UMI, N=32) and non-enriched libraries (i.e., Metagenome and Metagenome-UMI, N=32) were conducted using limma (Ritchie *et al.*, 2015), adjusting for multiple comparisons using the Benjamini-Hochberg procedure and a critical α of 0.05 (Benjamini and Hochberg, 1995).

To evaluate the ability of each library preparation assay to provide complete and uniform sequence data across targets, the following metrics were compared between each group: the depth of coverage (defined as the average number of aligned reads per nucleotide within the sequence), breadth of coverage (defined as the proportion of nucleotides within a sequence aligned by at least 1 read), and evenness of coverage as measured using Shannon Entropy and L^2^-norm deviation across sequences (Shannon, 1948). Shannon Entropy and L^2^-norm deviation were calculated with a normalized vector (obtained by summing the vector of nucleotides to 1.0), such that indices across sequences were comparable (i.e., for all sequences, the indices ranged from 0 to 1). For these comparisons, all genes and all reads were included in the analysis (not just genes and reads that reached the 80% gene fraction threshold). Comparisons were made using linear mixed models with assay and number of reinflated nonhost reads per sample as fixed effects and sample type and sample ID as random-slope and random-intercept effects; slopes of sample ID were allowed to vary by assay (Smyth, 2004). The significance of assay on these coverage and skewness metrics was tested using a log-likelihood test comparing nested models with and without assay as a predictor variable, and Tukey all-pair contrasts between the 4 assays were conducted using generalized linear hypothesis tests to correct for multiple comparisons of means (Hothorn *et al.*, 2008). Coefficients for the sample type random effects were calculated as a combination of fixed effects and variance components, using the “coef” function of lme4 (Smyth, 2004).

To compare overall resistome composition across assays, ordination using non-metric multidimensional scaling (NMDS) was performed on the Euclidean distances between sequencing datasets after counts of alignments at the class level had been normalized with cumulative sum scaling (Paulson *et al.*, 2013) and Hellinger-transformed to account for uneven sequencing depth (Legendre and Gallagher, 2001). The number of ordination dimensions was increased until the ordination stress was <0.05. Richness, Simpson’s diversity index and equitability were also compared between assays using linear mixed models with assay and the number of reinflated nonhost reads per sample as fixed effects, and sample type and sample ID as random effects with random-intercept and random-slopes effects (Smyth, 2004).

In order to identify potential drivers of library bias introduced by MEGaRICH, we analyzed associations between the proportion of Resistome-UMI families containing only one sequence (“singletons”, i.e., DNA sequences that were not amplified and/or not sequenced more than once), and sample-level characteristics for each of the 16 samples. Characteristics included inherent features of the sample (i.e., resistome richness and diversity), as well as library preparation and sequencing characteristics (i.e., 260/280 DNA absorbance ratios, proportion of DNA recovered after post-capture amplification, proportion of post-capture library included in pooling for sequencing, and the number of reads produced by sequencing). A descriptive scatterplot with R^2^ value was used explore univariable associations due to a lack of power (n=16 observations for the Resistome-UMI dataset, see Fig. S1 and S2).

*References in Supplementary Methods*

Benjamini Y, Hochberg Y. (1995). Controlling the false discovery rate: a practical and powerful approach to multiple testing. *J R Stat Soc Ser B* **57**: 289–300.

Bolger AM, Lohse M, Usadel B. (2014). Trimmomatic: A flexible trimmer for Illumina Sequence Data. *Bioinformatics* btu170.

Bush K, Jacoby GA. (2010). Updated functional classification of beta-lactamases. *Antimicrob Agents Chemother* **54**: 969–976.

Chen L, Xiong Z, Sun L, Yang J, Jin Q. (2012). VFDB 2012 update: toward the genetic diversity and molecular evolution of bacterial virulence factors. *Nucleic Acids Res* **40**: D641–645.

Chen L, Yang J, Yu J, Yao Z, Sun L, Shen Y, *et al.* (2005). VFDB: a reference database for bacterial virulence factors. *Nucleic Acids Res* **33**: D325–D328.

Cock PJA, Antao T, Chang JT, Chapman BA, Cox CJ, Dalke A, *et al.* (2009). Biopython: freely available Python tools for computational molecular biology and bioinformatics. *Bioinformatics* **25**: 1422–1423.

Edgar RC. (2010). Search and clustering orders of magnitude faster than BLAST. *Bioinformatics* **26**: 2460–2461.

Fu L, Niu B, Zhu Z, Wu S, Li W. (2012). CD-HIT: accelerated for clustering the next-generation sequencing data. *Bioinforma Oxf Engl* **28**: 3150–3152.

Garrison E, Marth G. (2012). Haplotype-based variant detection from short-read sequencing. *arXiv* **arXiv:1207.3907 [q-bio.GN]**.

Gibson MK, Forsberg KJ, Dantas G. (2015). Improved annotation of antibiotic resistance determinants reveals microbial resistomes cluster by ecology. *ISME J* **9**: 207–216.

Gupta SK, Padmanabhan BR, Diene SM, Lopez-Rojas R, Kempf M, Landraud L, *et al.* (2014). ARG-ANNOT, a new bioinformatic tool to discover antibiotic resistance genes in bacterial genomes. *Antimicrob Agents Chemother* **58**: 212–220.

Hothorn T, Bretz F, Westfall P. (2008). Simultaneous Inference in General Parametric Models. *Biom J* **50**: 346–363.

Kennedy SR, Schmitt MW, Fox EJ, Kohrn BF, Salk JJ, Ahn EH, *et al.* (2014). Detecting ultralow-frequency mutations by Duplex Sequencing. *Nat Protoc* **9**: 2586–2606.

Kent WJ. (2002). BLAT—The BLAST-Like Alignment Tool. *Genome Res* **12**: 656–664.

Lakin SM, Dean C, Noyes NR, Dettenwanger A, Spencer Ross A, Doster E, *et al.* (2016). MEGARes: an antimicrobial resistance database for high throughput sequencing. *Nucleic Acids Res* 1–7.

Legendre P, Gallagher ED. (2001). Ecologically meaningful transformations for ordination of species data. *Oecologia* **129**: 271–280.

Li H, Durbin R. (2009). Fast and accurate short read alignment with Burrows–Wheeler transform. *Bioinformatics* **25**: 1754–1760.

Li H, Handsaker B, Wysoker A, Fennell T, Ruan J, Homer N, *et al.* (2009). The Sequence Alignment/Map format and SAMtools. *Bioinforma Oxf Engl* **25**: 2078–2079.

McArthur AG, Waglechner N, Nizam F, Yan A, Azad MA, Baylay AJ, *et al.* (2013). The Comprehensive Antibiotic Resistance Database. *Antimicrob Agents Chemother* **57**: 3348–3357.

Morgulis A, Gertz EM, Schäffer AA, Agarwala R. (2006). A fast and symmetric DUST implementation to mask low-complexity DNA sequences. *J Comput Biol J Comput Mol Cell Biol* **13**: 1028–1040.

Pal C, Bengtsson-Palme J, Rensing C, Kristiansson E, Larsson DGJ. (2014). BacMet: antibacterial biocide and metal resistance genes database. *Nucleic Acids Res* **42**: D737–743.

Paulson JN, Stine OC, Bravo HC, Pop M. (2013). Differential abundance analysis for microbial marker-gene surveys. *Nat Methods* **10**: 1200–1202.

Ritchie ME, Phipson B, Wu D, Hu Y, Law CW, Shi W, *et al.* (2015). limma powers differential expression analyses for RNA-sequencing and microarray studies. *Nucleic Acids Res* gkv007.

Shannon C. (1948). A Mathematical Theory of Communication. *Bell Syst Tech J* **27**: 379–423,623–656.

Smyth GK. (2004). Linear models and empirical bayes methods for assessing differential expression in microarray experiments. *Stat Appl Genet Mol Biol* **3**: Article3.

Wood DE, Salzberg SL. (2014). Kraken: ultrafast metagenomic sequence classification using exact alignments. *Genome Biol* **15**: R46.

Zankari E, Hasman H, Cosentino S, Vestergaard M, Rasmussen S, Lund O, *et al.* (2012). Identification of acquired antimicrobial resistance genes. *J Antimicrob Chemother* **67**: 2640–2644.
