# Supplementary material for: Enrichment allows identification of diverse, rare elements in metagenomic resistome-virulome sequencing
Source: Microbiome. 2017 Oct 17;5:142. doi: 10.1186/s40168-017-0361-8 (PMC5645900; doi:10.1186/s40168-017-0361-8)
Supplement: Supplementary file 4 — Number of aligned reads to each gene group identified only in enriched samples, by resistance class and sample type. (DOCX 41 kb) [file 40168_2017_361_MOESM4_ESM.docx]

Table S1. Number of aligned reads to each gene group that was identified only in enriched samples, by class and sample source. VF = virulence factor

| **Class** | **Group** | **Beef** | **Poultry** | **Swine** | **WWTP** |
| --- | --- | --- | --- | --- | --- |
| Acid resistance | eefA | 0 | 256 | 37 | 1411 |
| Acid resistance | eefX | 0 | 88 | 7 | 488 |
| Adherence and Invasion (VF) | N/A | 24824 | 120666 | 276292 | 7448 |
| Aluminum resistance | queC | 0 | 0 | 0 | 8 |
| Aminoglycosides | AAC2-PRIME | 0 | 0 | 0 | 12 |
| Aminoglycosides | AAC3-PRIME | 0 | 0 | 0 | 414 |
| Aminoglycosides | RMTD | 0 | 0 | 41 | 0 |
| Aminoglycosides | RMTG | 0 | 0 | 199 | 0 |
| Aminoglycosides | SPD | 0 | 868 | 48 | 0 |
| Bacitracin | BCRB | 31 | 0 | 0 | 0 |
| Bacitracin | BCRC | 25 | 0 | 0 | 0 |
| betalactams | ACT | 0 | 15 | 0 | 1267 |
| betalactams | AER | 0 | 0 | 0 | 58689 |
| betalactams | AIM | 0 | 0 | 0 | 6515 |
| betalactams | BLA1 | 0 | 0 | 0 | 74 |
| betalactams | BLAA | 0 | 0 | 0 | 34 |
| betalactams | BLAI | 5 | 0 | 0 | 0 |
| betalactams | BLAR | 75 | 0 | 0 | 0 |
| betalactams | BLAZ | 0 | 263 | 312 | 547 |
| betalactams | CARB | 0 | 0 | 2614 | 376687 |
| betalactams | CEPA | 15 | 21631 | 12871 | 80 |
| betalactams | CFIA | 0 | 34059 | 0 | 0 |
| betalactams | CMY | 42 | 4599 | 0 | 1783 |
| betalactams | GES | 0 | 0 | 0 | 3270887 |
| betalactams | KPC | 0 | 0 | 0 | 717 |
| betalactams | MECA | 0 | 0 | 252 | 88 |
| betalactams | MECI | 0 | 0 | 0 | 30 |
| betalactams | MIR | 0 | 0 | 0 | 189 |
| betalactams | MOX | 0 | 0 | 0 | 252 |
| betalactams | OKP | 0 | 0 | 0 | 112 |
| betalactams | OXY | 0 | 0 | 0 | 10 |
| betalactams | PBP1B | 0 | 0 | 0 | 1535 |
| betalactams | PBP2B | 0 | 0 | 0 | 71045 |
| betalactams | ROB | 103457 | 244 | 21744 | 0 |
| betalactams | SCO | 0 | 0 | 0 | 2786 |
| betalactams | TEM | 180017 | 534985 | 467609 | 20544 |
| betalactams | TLA | 0 | 0 | 0 | 39858 |
| betalactams | VEB | 0 | 0 | 0 | 17204 |
| Biguanide resistance | CEPA | 0 | 201 | 80 | 1328 |
| Biocide resistance | ebrA | 7 | 0 | 0 | 0 |
| Biocide resistance | ebrB | 6 | 0 | 0 | 0 |
| Biocide resistance | emmdR | 0 | 0 | 0 | 403 |
| Biocide resistance | OmpD | 0 | 392 | 0 | 0 |
| Biocide resistance | oxyR | 0 | 264 | 0 | 2086 |
| Biocide resistance | phoB | 0 | 140 | 14 | 797 |
| Biocide resistance | phoR | 0 | 488 | 47 | 2351 |
| Biocide resistance | smvA | 0 | 89 | 0 | 0 |
| Chromium resistance | chrA | 0 | 0 | 0 | 35481 |
| Chromium resistance | chrA1 | 0 | 0 | 0 | 31 |
| Chromium resistance | chrE | 0 | 0 | 0 | 7 |
| Chromium resistance | chrF | 0 | 0 | 0 | 57 |
| Copper resistance | copC | 0 | 8 | 47 | 1420 |
| Copper resistance | copJ | 0 | 0 | 0 | 10 |
| Copper resistance | copK | 0 | 0 | 0 | 88 |
| Copper resistance | copL | 0 | 0 | 0 | 2326 |
| Copper resistance | csoR | 11 | 0 | 0 | 28 |
| Copper resistance | cueP | 0 | 351 | 0 | 0 |
| Copper resistance | ycnK | 15 | 0 | 0 | 18 |
| Copper resistance | yfmP | 14 | 0 | 0 | 0 |
| Drug and biocide resistance | adeB | 0 | 0 | 0 | 396 |
| Drug and biocide resistance | ADEF | 0 | 0 | 0 | 117 |
| Drug and biocide resistance | ADEG | 0 | 0 | 0 | 877 |
| Drug and biocide resistance | adeH | 0 | 0 | 0 | 407 |
| Drug and biocide resistance | adeI | 0 | 0 | 0 | 247 |
| Drug and biocide resistance | adeK | 0 | 0 | 0 | 48 |
| Drug and biocide resistance | adeL | 0 | 0 | 0 | 565 |
| Drug and biocide resistance | ADEN | 0 | 0 | 0 | 29 |
| Drug and biocide resistance | adeT1 | 0 | 0 | 0 | 72 |
| Drug and biocide resistance | adeT2 | 0 | 0 | 0 | 205 |
| Drug and biocide resistance | amvA | 0 | 0 | 0 | 435 |
| Drug and biocide resistance | bexA | 0 | 3553 | 0 | 1308 |
| Drug and biocide resistance | bmrR | 20 | 0 | 0 | 24 |
| Drug and biocide resistance | cpxA | 0 | 179 | 0 | 1348 |
| Drug and biocide resistance | cpxR | 0 | 98 | 0 | 841 |
| Drug and biocide resistance | emrBsm | 0 | 0 | 0 | 139 |
| Drug and biocide resistance | emrRsm | 0 | 0 | 0 | 3 |
| Drug and biocide resistance | kexD | 0 | 86 | 137 | 5089 |
| Drug and biocide resistance | mdfA | 0 | 154 | 0 | 666 |
| Drug and biocide resistance | mdtG | 0 | 0 | 0 | 582 |
| Drug and biocide resistance | mdtI | 0 | 23 | 0 | 46 |
| Drug and biocide resistance | mdtJ | 0 | 132 | 16 | 435 |
| Drug and biocide resistance | norM | 0 | 0 | 0 | 805 |
| Drug and biocide resistance | ompC | 0 | 838 | 136 | 3234 |
| Drug and biocide resistance | OQXA | 0 | 0 | 34 | 962 |
| Drug and biocide resistance | oqxB | 0 | 212 | 0 | 1002 |
| Drug and biocide resistance | qacB | 0 | 0 | 0 | 2195 |
| Drug and biocide resistance | qacJ | 0 | 0 | 0 | 14 |
| Drug and biocide resistance | qacR | 0 | 0 | 0 | 309 |
| Drug and biocide resistance | tolC | 16960 | 153875 | 58153 | 0 |
| Drug, metal and biocide resistance | baeS | 0 | 49 | 0 | 0 |
| Drug, metal and biocide resistance | cmeA | 141 | 256 | 0 | 0 |
| Drug, metal and biocide resistance | cmeC | 228 | 209 | 0 | 0 |
| Drug, metal and biocide resistance | gesA | 0 | 121 | 0 | 0 |
| Drug, metal and biocide resistance | gesB | 0 | 709 | 0 | 0 |
| Drug, metal and biocide resistance | gesC | 0 | 175 | 0 | 0 |
| Drug, metal and biocide resistance | mdtA | 0 | 91 | 0 | 0 |
| Drug, metal and biocide resistance | mdtC | 0 | 73 | 0 | 0 |
| Fluoroquinolones | NORA | 0 | 0 | 24 | 40 |
| Fluoroquinolones | QNR | 0 | 0 | 0 | 27 |
| Fluoroquinolones | QNRA | 0 | 0 | 0 | 241 |
| Fluoroquinolones | QNRB | 0 | 0 | 0 | 15454 |
| Fluoroquinolones | QNRS | 0 | 0 | 0 | 7212 |
| Fluoroquinolones | QNRVC | 0 | 0 | 0 | 12945 |
| Fosfomycin | FOSA | 0 | 38 | 17 | 406 |
| Fosfomycin | FOSX | 0 | 0 | 0 | 18 |
| Glycopeptides | VANA | 0 | 0 | 0 | 965 |
| Glycopeptides | VANB | 0 | 0 | 1690 | 59 |
| Glycopeptides | VANC | 0 | 729 | 0 | 45 |
| Glycopeptides | VAND | 0 | 0 | 0 | 210 |
| Glycopeptides | VANG | 0 | 0 | 3729 | 1744 |
| Glycopeptides | VANHA | 0 | 67 | 0 | 364 |
| Glycopeptides | VANHB | 0 | 0 | 1346 | 202 |
| Glycopeptides | VANHD | 0 | 0 | 0 | 33 |
| Glycopeptides | VANRA | 0 | 23 | 0 | 590 |
| Glycopeptides | VANRB | 0 | 0 | 1119 | 232 |
| Glycopeptides | VANRC | 0 | 279 | 0 | 94 |
| Glycopeptides | VANRD | 0 | 0 | 0 | 226 |
| Glycopeptides | VANRG | 0 | 0 | 0 | 165 |
| Glycopeptides | VANSA | 0 | 90 | 0 | 707 |
| Glycopeptides | VANSB | 0 | 0 | 2233 | 177 |
| Glycopeptides | VANSC | 0 | 729 | 0 | 274 |
| Glycopeptides | VANSD | 0 | 0 | 0 | 616 |
| Glycopeptides | VANSG | 0 | 0 | 0 | 315 |
| Glycopeptides | VANTC | 0 | 1602 | 0 | 160 |
| Glycopeptides | VANTG | 0 | 0 | 11932 | 7217 |
| Glycopeptides | VANUG | 0 | 0 | 0 | 12 |
| Glycopeptides | VANWA | 0 | 416 | 0 | 0 |
| Glycopeptides | VANWB | 0 | 0 | 1308 | 51 |
| Glycopeptides | VANWG | 0 | 0 | 122 | 613 |
| Glycopeptides | VANXA | 0 | 39 | 0 | 530 |
| Glycopeptides | VANXB | 0 | 0 | 1508 | 157 |
| Glycopeptides | VANXD | 0 | 0 | 0 | 103 |
| Glycopeptides | VANXYC | 0 | 165 | 0 | 56 |
| Glycopeptides | VANXYG | 0 | 0 | 2877 | 2233 |
| Glycopeptides | VANYA | 0 | 0 | 0 | 891 |
| Glycopeptides | VANYB | 0 | 0 | 1175 | 278 |
| Glycopeptides | VANYD | 0 | 0 | 0 | 25 |
| Glycopeptides | VANYG | 0 | 0 | 0 | 256 |
| Glycopeptides | VANZA | 0 | 0 | 0 | 159 |
| Gold resistance | golS | 0 | 41 | 0 | 0 |
| Intracellular Survival and Growth (VF) | N/A | 0 | 829 | 0 | 0 |
| Iron acquisition (VF) | N/A | 8792 | 598747 | 69894 | 518 |
| Iron resistance | pmrG | 0 | 197 | 0 | 0 |
| Lead resistance | pbrD | 0 | 0 | 0 | 44 |
| Magnesium resistance | mgtA | 0 | 1256 | 0 | 0 |
| Manganese resistance | mntA | 39 | 0 | 0 | 62 |
| Mercury resistance | merF | 0 | 303 | 7 | 35200 |
| Mercury resistance | merG | 0 | 0 | 0 | 276 |
| Mercury resistance | merH | 0 | 0 | 0 | 63 |
| Mercury resistance | merR2 | 0 | 28 | 0 | 8428 |
| Metal and biocide resistance | ABEM | 0 | 0 | 0 | 310 |
| Metal and biocide resistance | chrC | 0 | 0 | 13 | 20 |
| Metal and biocide resistance | chrF | 0 | 0 | 7 | 45 |
| Metal and biocide resistance | cueO | 0 | 129 | 0 | 0 |
| Metal and biocide resistance | dpr | 0 | 83 | 98 | 42510 |
| Metal and biocide resistance | sitA | 0 | 395 | 0 | 0 |
| Metal and biocide resistance | sitB | 0 | 447 | 0 | 0 |
| Metal and biocide resistance | sitC | 0 | 329 | 0 | 0 |
| Metal and biocide resistance | sitD | 0 | 504 | 0 | 0 |
| Metal resistance | corA | 442 | 2200 | 1566 | 0 |
| Metal resistance | corB | 0 | 377 | 0 | 0 |
| Metal resistance | corC | 0 | 2759 | 0 | 0 |
| Metal resistance | corD | 0 | 157 | 0 | 0 |
| Metal resistance | czcA | 8893 | 1537 | 3589 | 110 |
| Metal resistance | czcB | 3168 | 587 | 1308 | 52 |
| Metal resistance | czcC | 2265 | 421 | 878 | 0 |
| Metal resistance | czcD | 4006 | 680 | 1508 | 228 |
| Metal resistance | czcE | 708 | 166 | 281 | 34 |
| Metal resistance | czcP | 0 | 0 | 0 | 140 |
| Metal resistance | czcR | 3242 | 596 | 1272 | 146 |
| Metal resistance | czcS | 3860 | 695 | 1397 | 0 |
| Metal resistance | golT | 0 | 525 | 0 | 0 |
| Metal resistance | modA | 0 | 0 | 0 | 326 |
| Metal resistance | modB | 0 | 0 | 0 | 111 |
| Metal resistance | nreB | 0 | 0 | 0 | 88 |
| Metal resistance | tunR | 0 | 0 | 0 | 217 |
| Metronidazole | NIMD | 0 | 0 | 0 | 331 |
| Metronidazole | NIME | 0 | 0 | 0 | 13 |
| Macrolide-Lincosamide-Streprogramin | EREB | 0 | 0 | 0 | 44332 |
| Macrolide-Lincosamide-Streprogramin | ERM41 | 0 | 0 | 0 | 7 |
| Macrolide-Lincosamide-Streprogramin | ERM42 | 0 | 0 | 103 | 624 |
| Macrolide-Lincosamide-Streprogramin | LNUF | 0 | 21 | 3161 | 67519 |
| Macrolide-Lincosamide-Streprogramin | MEFB | 0 | 27 | 0 | 247613 |
| Macrolide-Lincosamide-Streprogramin | MPHB | 435 | 0 | 0 | 0 |
| Macrolide-Lincosamide-Streprogramin | MSRA | 0 | 10621 | 186 | 2019 |
| Macrolide-Lincosamide-Streprogramin | MSRC | 0 | 19898 | 0 | 3836 |
| Macrolide-Lincosamide-Streprogramin | VATC | 0 | 4905 | 0 | 0 |
| Macrolide-Lincosamide-Streprogramin | VGBB | 0 | 521 | 0 | 0 |
| Multi-drug resistance | ABEM | 0 | 0 | 0 | 485 |
| Multi-drug resistance | ACRB | 0 | 0 | 0 | 3535 |
| Multi-drug resistance | adeB | 0 | 0 | 0 | 2602 |
| Multi-drug resistance | ADEF | 0 | 0 | 0 | 82 |
| Multi-drug resistance | ADEG | 0 | 0 | 0 | 579 |
| Multi-drug resistance | adeH | 0 | 0 | 0 | 393 |
| Multi-drug resistance | ADEN | 0 | 0 | 0 | 144 |
| Multi-drug resistance | ADER | 0 | 0 | 0 | 104 |
| Multi-drug resistance | cme | 154 | 0 | 0 | 0 |
| Multi-drug resistance | EMEA | 0 | 2748 | 3125 | 647 |
| Multi-drug resistance | EMRD | 0 | 117 | 0 | 295 |
| Multi-drug resistance | golS | 0 | 39 | 0 | 0 |
| Multi-drug resistance | MDSA | 0 | 113 | 0 | 0 |
| Multi-drug resistance | MDSB | 0 | 690 | 0 | 0 |
| Multi-drug resistance | MDSC | 0 | 75 | 0 | 0 |
| Multi-drug resistance | MEPR | 0 | 0 | 0 | 18 |
| Multi-drug resistance | MEXE | 0 | 0 | 0 | 1584 |
| Multi-drug resistance | MEXT | 0 | 0 | 0 | 121 |
| Multi-drug resistance | MSBA | 0 | 0 | 0 | 21256 |
| Multi-drug resistance | OQXA | 0 | 148 | 0 | 513 |
| Multi-drug resistance | oqxB | 0 | 369 | 0 | 1977 |
| Multi-drug resistance | QACAB | 0 | 0 | 145 | 3240 |
| Multi-drug resistance | RAMA | 0 | 0 | 0 | 54 |
| Multi-drug resistance | ROBA | 0 | 0 | 0 | 432 |
| Multi-drug resistance | SAV1866 | 0 | 0 | 0 | 62 |
| Multi-drug resistance | SDIA | 0 | 267 | 0 | 0 |
| Multi-drug resistance | YKKC | 16 | 0 | 0 | 0 |
| Multi-drug resistance | YKKD | 0 | 0 | 0 | 12 |
| Nickel resistance | ncrA | 0 | 0 | 0 | 2149 |
| Nickel resistance | ncrB | 0 | 0 | 0 | 711 |
| Nickel resistance | ncrC | 0 | 0 | 0 | 1794 |
| Nickel resistance | ncrY | 0 | 0 | 0 | 956 |
| Nickel resistance | nirB | 0 | 0 | 0 | 82 |
| Nickel resistance | nirD | 0 | 0 | 0 | 20 |
| Paraquat resistance | yddg | 0 | 232 | 0 | 0 |
| Peroxide resistance | sodB | 0 | 0 | 0 | 75 |
| Phenicol | CATA | 0 | 5138 | 1778 | 36 |
| Phenicol | CATB | 647 | 0 | 18 | 13307 |
| Phenicol | CATP | 2497 | 3583 | 536 | 12040 |
| Phenicol | CFRA | 2050 | 0 | 433 | 0 |
| Phenicol | CLBC | 189 | 0 | 0 | 0 |
| Phenicol | CML | 0 | 0 | 114154 | 50006 |
| Phenicol | FEXA | 35488 | 0 | 66 | 0 |
| Phenolic compound resistance | fabL | 34 | 0 | 0 | 36 |
| Regulation of Virulence (VF) | N/A | 534 | 14353 | 3425 | 76 |
| Rifampin | ARR | 0 | 0 | 0 | 839 |
| Secretion System (VF) | N/A | 4286 | 122236 | 427295 | 8658 |
| Tellurium resistance | terB | 0 | 809 | 0 | 567 |
| Tellurium resistance | terC | 0 | 5030 | 0 | 3296 |
| Tellurium resistance | terD | 0 | 2278 | 0 | 1161 |
| Tellurium resistance | terE | 0 | 2334 | 0 | 1756 |
| Tetracyclines | TET31 | 0 | 0 | 2260 | 1751 |
| Tetracyclines | TET36 | 324 | 51 | 418 | 2894 |
| Tetracyclines | TET39 | 0 | 331 | 2422 | 100284 |
| Tetracyclines | TETE | 0 | 0 | 0 | 6019 |
| Tetracyclines | TETG | 9545 | 2630 | 4607 | 41174 |
| Tetracyclines | TETH | 258 | 5409 | 13606 | 32 |
| Tetracyclines | TETJ | 0 | 12643 | 0 | 0 |
| Tetracyclines | TETS | 1723 | 1954 | 9563 | 21303 |
| Tetracyclines | TETT | 14380 | 42590 | 9229 | 9889 |
| Tetracyclines | TETY | 0 | 0 | 9272 | 45 |
| Toxin Production (VF) | N/A | 8480 | 552 | 6521 | 432 |
| Trimethoprim | DFRA | 76 | 1122 | 20 | 2722 |
| Trimethoprim | DFRB | 0 | 0 | 0 | 91 |
| Trimethoprim | DFRK | 0 | 121 | 0 | 0 |
