# Supplementary material for: Enrichment allows identification of diverse, rare elements in metagenomic resistome-virulome sequencing
Source: Microbiome. 2017 Oct 17;5:142. doi: 10.1186/s40168-017-0361-8 (PMC5645900; doi:10.1186/s40168-017-0361-8)
Supplement: Supplementary file 13 — Primers used in custom dual-indexed UMI protocol. (DOCX 68 kb) [file 40168_2017_361_MOESM13_ESM.docx]

|  | **Purpose** | **Name** | **Sequence** | **Modifications from Original Sequence** |
| --- | --- | --- | --- | --- |
| Adapter Oligos | Used to create UMI adapters | MWS51_KJ | 5′-ACACTCTTTCCCTACACGACGCTCTTCCGATC-3′ | MWS51 with 25 bp removed 5’ and 1 bp removed 3’ |
|  |  | MWS55_KJ | 5′-TCTTCTACAGTCANNNNNNNNNNNNAGATCG GAAGAGCACACGTCGGAACTCCAGTCAC-3′ | MWS55 with base 46 changed from T to G |
| Index Primers | Used to produce dual-indexed libraries | MWS21_KJ_i7 | 5′-CAAGCAGAAGACGGCATACGAGAT[i7-XXXXXXXX]GTGACTGGAGTTCCGACGTGTGC-3′ | MWS21 with index length increased from 6 bp to 8 bp; base 14 after index changed from A to C |
|  |  | MWS51+_i5 | 5′-AATGATACGGCGACCACCGAGATCTACAC[i5-XXXXXXXX]ACACTCTTTCCCTACACGAC-3′ | MWS51 5’ 29 bp, followed by 8 bp index, followed by MWS51 base 26 to base 45 |
| Post-Capture Primers | Used to amplify libraries after target capture | MWS13 | 5′-AATGATACGGCGACCACCGAG-3′ | N/A |
|  |  | BP2 | 5’-CAAGCAGAAGACGGCATACGAG-3' | MWS21 5’ 22 bp |

Table S2. Primers used in custom dual-indexed UMI protocol
